# Electronic health record system in the public health care sector of South Africa: A systematic literature review

**DOI:** 10.4102/phcfm.v10i1.1746

**Published:** 2018-11-20

**Authors:** Munyaradzi C. Katurura, Liezel Cilliers

**Affiliations:** 1 Department of Information Systems, University of Fort Hare, South Africa

## Abstract

**Background:**

South Africa is planning to implement the National Health Insurance (NHI) scheme in the near future. The NHI is intended to improve the accessibility of quality health care services for all South African citizens. For the NHI to achieve this objective, an electronic health record (EHR) system to register and track patients who visit different health care providers will have to be developed.

**Aim:**

To identify critical success factors for the implementation of EHRs in South Africa’s public health care sector.

**Setting:**

This study reviewed studies on EHR implementation in African countries published between 2006 and 2017.

**Methods:**

The study made use of a systematic literature review to identify barriers to the implementation of EHRs in the public health care sector.

**Results:**

Fifteen articles were included in the study. The study identified technical, social and environmental barriers to the implementation of EHRs. The barriers could further be broken down into lack of supporting infrastructure; user training and commitment; political influence or strategy; legislation and regulations; and the lack of a framework for implementation and management of EHRs. The study suggests six main recommendations for the successful implementation of EHRs in South Africa’s public health care sector.

**Conclusion:**

The study recommended investing in alternative infrastructure facilities, incentivising the health informatics field to attract and retain information and communication technology professionals and to encourage the participation of all stakeholders in the development process to develop context-relevant e-health implementation strategies, legislation and frameworks. Government should also allocate separate budgets for e-health projects.

## Background

South Africa is developing many strategies towards improving the quality of public health care services as well as improving the accessibility of these services.^1^ One of these strategies is the implementation of National Health Insurance (NHI). The NHI is a health insurance scheme that is intended to provide health insurance coverage for all citizens of South Africa.^2^ The scheme will pay for patients’ visits to contracted private and public health care providers through a centralised reserve that will be funded through income tax. The NHI will allow citizens to access quality health care services regardless of their financial status.^1^

For the NHI to work efficiently, the scheme must be able to register and track patients. The National Department of Health, the Department of Science and Technology and the Council for Scientific and Industrial Research (CSIR) developed a patient registration system that could be used together with an electronic health record (EHR). The goal of this system is to allow patient tracking wherever patients present themselves in South Africa while solving the issues of lack of interoperability, fragmentation and the absence of a National Patient Master Index. The implementation of EHRs can be used to facilitate the registration and tracking of patients to improve the quality and continuity of care. Electronic health records enable sharing of patient data between points of care with the added advantage that current information is available to make decisions about health outcomes for health care providers.^3^ The EHR systems allow for the capturing, storing and sharing of a patient’s personal as well as medical information.^4^

However, African countries have not been successful in their implementations of EHRs.^4,5,6^ Both Malawi and Ghana have made attempts to implement a national EHR system, but challenges such as a lack of government support and necessary infrastructure, unavailability of a continuous electricity supply and resistance from health care workers caused these projects to be unsuccessful.^6,7,8^ In South Africa, the implementation of different EHR systems from various vendors presents a challenge as these systems are built with different underlying database architectures and therefore often fail to communicate and share information among each other. Also, while these systems have been implemented in some areas, more than half of the public health centres in South Africa still make use of a paper-based filing system.^4,8,9,10^

### Aim

This study sought to identify critical success factors (CSFs) for the implementation of EHRs in South Africa’s public health care sector. To accomplish this, literature on the implementation of EHRs in African countries’ public health care sectors was reviewed to identify the common barriers to the successful implementation of these systems. These findings were used to develop CSFs for the implementation of EHRs in South Africa’s public health care sector.

## Research methodology

This study made use of a qualitative, interpretive approach. The data was collected through a systematic literature review that followed the guidelines of the hermeneutic framework. This framework was developed based on the hermeneutic principle as discussed in the Cochrane Collaboration. The framework was chosen because it was suitable for conducting a qualitative synthesis of data and also allowed the researcher to conduct several iterations through the data sources to gain a better understanding as well as identify all relevant information to the study.^11,12^

The theoretical background for this study was the socio-technical theory. This theory was chosen for this study as it focuses on the implementation of technology in the organisation. The socio-technical theory focuses on the implementation of technology in organisations. There are social and technical elements found in organisations that influence the success or failure of technology implementation.^13,14^ The technical subsystem consists of the tools, devices and techniques necessary for transforming inputs into outputs in ways that make the organisation more efficient. The social subsystem consists of employees, needs, attitudes, skills, values and the knowledge they bring to the work environment, together with the reward system and structures of authority in the organisation.^11,15^

However, studies in the field of health informatics indicated that this theory does not cover all the factors that affect technology implementation in the health care field as there are environmental factors like rules and regulations that also affect the success of technology.^14,16^ This study therefore used the modified version of this theory that considers social, technical and environmental factors.

The literature that was analysed was sourced from Google Scholar, Science Direct, the PubMed Library, Scholar Droid, ResearchGate and the Association for Computing Machinery (ACM). An exhaustive search on the selected sources to identify all articles that fit the inclusion criteria was undertaken. The search included all articles that met the inclusion criteria regardless of the type of studies they were derived from. The study included all types of articles as long as they have addressed implementation of EHR in public health care. The context of the articles had to be African because the study wanted to consider examples from countries that were lower in terms of development thereby offering comparable circumstances to some of the rural and remote areas of South Africa. The articles were published between 2006 and 2017 to accommodate for the slow adoption of technology in Africa. A forward and backward search strategy was adopted to ensure that all useful references were included in the review. The strategy was implemented in such a way that if a relevant source had been identified, any sources that were cited in that study were also checked, thus completing the backwards step. The forwards step was completed by searching any sources that cited the identified relevant source. A database of the various keywords used in the search process was compiled detailing the multiple keywords and the reasons they were included in the search process. An extract of the keywords database is shown in Table 1. The full articles were downloaded from the various databases and analysed independently by two researchers. After the manual analysis was completed, the researchers compared their results in order to reach a consensus as to which of the articles should be included in the study.

**TABLE 1:** Keywords database extract.

Search phrase	Reason for modifying
Electronic health records (EHRs)	The original phrase used to search all databases
Electronic health records + implementation	Used to refine the search results to only include articles that focus on the implementation of EHRs
Electronic health records + public health	Used to eliminate studies that discussed EHR implementation in private health care providers
Electronic health records + public health + South Africa	Used to find studies that were conducted in South Africa only
Electronic health records + implementation + in Africa	Used to refine the search results to articles that focus on EHRs implementation in Africa
Electronic health records + implementation + in Africa/(name of African country)	Names of African countries known to have implemented EHRs such as Ghana, Nigeria and Kenya were used in the search query to refine the search
Electronic health records + public health + (name of African country)	Variations of the above search phrase used for identifying articles that did not talk about implementation process but discussed possible causes of project failures in African countries
Electronic Patient Record + implementation	A variation of EHR used in some sources referring to the same type of EHR system as defined in the study
Electronic Medical Record + implement	A variation of EHR used in some sources referring to the same type of EHR system as defined in the study
Computerised Patient Record + implement	A variation of EHR used in some sources referring to the same type of EHR system as defined in the study

EHR, electronic health record.

After collecting all the relevant information, a thematic analysis of the collected articles was carried out to identify recurrent themes in the articles. The final findings from the analysis of the gathered literature were grouped based on the themes found from the step above. The articles that were analysed were based on various types of studies and it was difficult to utilise any special tool in the data extraction process; therefore, the writers manually extracted and tabulated the data separately before comparing and combining them. After grouping the findings under each theme, they were synthesised into more relevant data for the current study by using the reciprocal translation method.^13^ In the reciprocal method, the findings of one study were compared to another and similarities were drawn from there, and the synthesised outcome was then compared to another study until all the articles had been synthesised. The results of the literature analysis were grouped into the three categories of factors outlined in the theoretical background.

### Ethical considerations

Ethical clearance to conduct the study was obtained from the University of Fort Hare, Research Ethics Committee (CIL041SKAT01).

## Results

**FIGURE 1:**
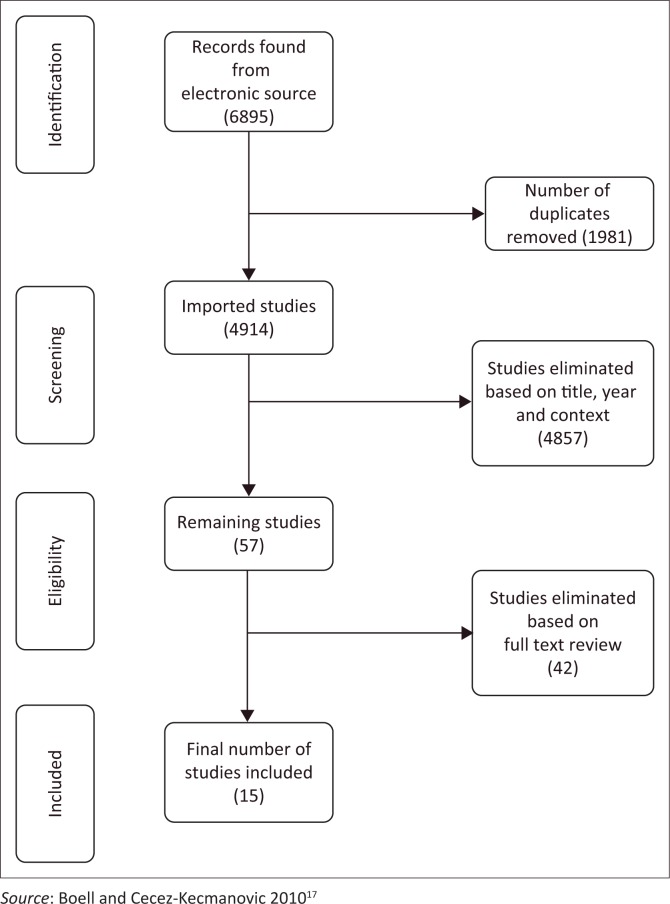
Adapted version of the PRISMA model representing all the steps of the data collection process.

The adapted Preferred Reporting Items for Systematic Reviews and Meta-Analysis (PRISMA) model in Figure 1^17^ shows the data collection process. The inclusion criteria included that the study must address EHRs implementation in the African public health context. The methodology used in the articles were not used as an exclusion or inclusion criteria, but ranged from opinion pieces and guidelines to Delphi studies seeking consensus. This decision was taken to enable a wide variety of studies to be included in the review. The search yielded a total of 6895 results from all the databases combined. However, these results included unsuitable and unfiltered data, so various methods such as search engine filtering, range and regional filtering were used to filter the results until 57 articles were left. These articles were read and then screened again for eligibility based on the following exclusion criteria: published before 2006 or after 2017 and whether the articles addressed EHR implementation in the private, not public, health sector in countries outside of Africa.

Fifty-seven articles were identified after the initial screening. The full text of these articles was reviewed, which reduced the final number to 15 articles. The 42 articles were eliminated for the following reasons:

- Twenty articles discussed EHR implementation but not in an Africa context.
- Seven articles were excluded because they focused on implementation of EHR in developing countries but failed to put context to any particular African country.
- Twelve articles did not address the implementation of EHR.
- Three articles could not be acquired in full text.

Table 2 shows a summary of the final selection of articles that were used in this study.

**TABLE 2:** Articles included in the study.

No.	Author	Title	Location	Year
1	Yogeswaran & Wright^18^	EHR implementation in South Africa: How do we get it right?	South Africa	2010
2	Weeks^19^	Electronic health records: Managing the transformation from a paper-based to an electronic system.	South Africa	2013
3	Thompson, Castle, Lubeck & Makarfi^20^	Experience implementing OpenMRS to support maternal and reproductive health in northern Nigeria.	Nigeria	2010
4	Ojo & Popoola^21^	Some correlates of electronic health information management system success in Nigerian teaching hospitals.	Nigeria	2015
5	Mugo & Nzuki^22^	Determinants of electronic health in developing countries.	Developing countries	2014
6	Mostert-Phipps, Pottas & Korpela^23^	Guidelines to encourage the adoption and meaningful use of health information technologies in the South African health care landscape.	South Africa	2013
7	Monawe, Chawani, Kapokosa & Moyo^5^	Strengthening health management information systems in Malawi: Gaps and opportunities.	Malawi	2015
8	Luna, Almerares, Mayan, de Quirós & Otero^24^	Health informatics in developing countries: Going beyond pilot practices to sustainable implementations: A review of the current challenges.	Developing countries	2014
9	Kusi^25^	Electronic health record system: A survey in Ghanaian hospitals.	Ghana	2012
10	Kleynhans^26^	Is South Africa ready for a national electronic health record (EHR)?	South Africa	2011
11	Juma, Nahason, Apollo, Gregory & Patrick^27^	Current status of e-health in Kenya and emerging global research trends.	Kenya	2012
13	Cline & Luiz^28^	Information technology systems in public sector health facilities in developing countries: The case of South Africa.	South Africa	2013
14	Sikhondze & Erasmus^29^	Electronic medical records: A developing and developed country analysis.	South Africa	2016
15	Akanbi, Ocheke, Agaba, Daniyam, Agaba, Okeke & Ukoli^4^	Use of electronic health records in sub-Saharan Africa: Progress and challenges.	Sub-Saharan Africa	2012

EHR, electronic health records.

## Social factors

There were two major themes in this category. Issues of user training, skills and commitment comprised the first theme under which the following factors were identified as challenges to the implementation of EHRs^5,27,30^:

- the lack of health care personnel with the necessary information and communication technology (ICT) skills to develop and maintain the EHR systems in the operational lifespan of the systems
- the lack of established career paths for health informatics specialists in the public health care sector
- resistance by health care personnel who felt that these systems would complicate work processes or even result in job losses because of failure to learn these systems.

The second theme identified was regarding issues of political influence. Under this theme the following factors were identified as challenges^21,23,26^:

- changes in government structures such as changes in presidency or cabinet ministers influenced the efforts to implement EHRs
- the occurrence of political unrest in individual countries has often resulted in inadequate commitment to the development of public services such as health care and all its related functions.

## Technical factors

The problems related to technical factors were divided into two major themes, which were lack of information technology (IT) infrastructure and cost of implementation. The issues that referred to the lack of IT infrastructure identified in literature included problems like:

- the lack of computers, printers and networking equipment for implementing EHRs as well as the uneven distribution of ICT hardware between urban areas and rural areas^4,20,24^
- the lack of Internet connectivity in some remote locations and a lack of stable electricity supply for supporting the systems were also noted in some literature.^4,5,27,28^

Issues related to the cost of implementing EHRs included^23,24,29^:

- the high costs of developing and implementing EHRs
- the lack of adequate funding from governments aimed at public health care development projects
- a lack of long-term financial commitment to fund the maintenance of these systems once implemented
- the poor management of donor funding that is directed towards the implementation of EHRs was identified as other capital challenges that hindered the implementation of EHRs in some African countries’ public health care systems.

## Environmental themes

The environmental problems that were found were grouped into three major theme categories, which were issues related to policies and strategies; legislations and regulations; and strategy and frameworks.

Problems found in literature that were relating to policies and strategies for the implementation of EHRs included the following:

- The various government policies and strategies that government implemented were lacking coordination and harmony, for example, the national ICT strategy might not be in line with the national e-health strategy in terms of objectives and goals of the strategy.^22,23,24^
- A second challenge was the lack of specificity in the national strategies as they would refer to e-health implementation in general, instead of specific systems such as telemedicine and EHRs.^13^

The main issue regarding legislation and regulation was that a lack of legislation and regulations regarding the implementation and use of EHRs promoted misuse and poor compliance to standards for any organisation.^21^ The lack of compliance had the effect of eroding patients’ trust in the EHR systems and their security. Government policies regarding restrictions, incentives, regulations and tax also influenced the success of EHR implementations in some of the literature.^22,24^

The issues found under the theme of standards and frameworks were that there was inadequate development of standard and frameworks for the implementation of EHRs in an African context. The lack of standards governing the development and implementation of EHRs resulted in systems from various vendors not being interoperable.^24,25^ In many cases, the systems were not developed and implemented following common guidelines that would enable them to share information and resources.^5,27,31^ The next section will discuss the recommendations of the study.

## Discussion and recommendations

The following factors were identified as critical to the successful implementation of EHRs in South Africa’s public health care sector based on the challenges identified in this study. There were six factors identified and for each of the factors recommendations of how these factors could be implemented were also given. Critical success factors are defined as ‘factors of a strategy that must be achieved to meet the objectives of the project’.^11^ While the content filtering services (CFSs) for each project may be unique, this study identified factors that are common in EHR implementation projects in African countries.

### Critical success factor 1: Incentivising the health informatics career field to attract and retain information and communication technology professionals

The public health care sector needs to attract and retain ICT professionals who can maintain the EHR systems once they are implemented to ensure that the systems remain operational. This factor aims to solve issues related to user training and commitment such as lack of qualified personnel to run and maintain the systems. The establishment of clear career paths with rewards and incentives for health informaticians can be a way of attracting ICT professionals to take up careers in the health care sector.^5,24^ Incentivising careers in the field could also motivate health care workers to learn how to use these EHR systems. Ojo and Popoola^18^ and Yogeswaran and Wright^20^ stated that offering incentives such as bonus salaries and promotions can motivate health care workers to participate in EHR training and implementation.

#### Recommendations regarding critical success factor 1

Incentivising the health informatics field has been noted as a way of promoting the development of the career field and retaining the expertise developed within. This study recommends the following strategies for incentivising health informatics career fields:

- Create a proper hierarchy for health informaticians similar to those found in the ICT fields (e.g. technicians, managers, supervisors and administrators), which provides clear opportunities for career advancement within the health informatics field.
- Offer pay grade reviews and upgrades for health care professionals who undertake training in line with EHR systems.

### Critical success factor 2: Encouraging participation of all stakeholders in the development process of electronic health record systems

All stakeholders including government, health care workers and EHR software developers need to work together to ensure the development of solutions that are suitable and scalable to suit the target organisation. The collaboration can also ensure that EHR systems are tailored to provide common work processes for easier transition for the health care workers who will use the systems. The involvement of health care professionals in the development process can increase their commitment to the success of the project.^25^

User participation in the development of EHR systems can lead to the development of a sense of ownership of the project and increased commitment to the project. Involving health care professionals in the development of EHR systems will minimise the need for training on how to use the final system. According to Mostert-Phipps et al.,^23^ the involvement of health care professionals in the development of their EHR systems will guarantee that the systems are made to resemble their current work processes, thereby ensuring that the work processes remain familiar to the workers, and where training is required it will be minimal.

Encouraging government to help improve the political influences by eliminating changes in government’s commitment to the project. However, this would require government to champion the implementation process and coordinate with all the stakeholders. Literature suggested that government should champion the implementation of all public services. It was proposed that government should not only be in charge of dispensing the financial resources but rather government as a stakeholder should also actively participate in the implementation of these services. Stable commitment from government will also mean sustained financial and legislative commitment to the implementation and maintenance of EHR projects at national level.^18,23^

#### Recommendations regarding critical success factor 2

The participation of all stakeholders in the development and implementation of EHRs is crucial as it affects the design and functionality of the EHRs. The participation of all stakeholders could also improve commitment of the stakeholders to the implementation and the overall success of the implementation. To implement this cooperation and collaboration from all stakeholders, the following recommendations were made:

- The government should develop a committee comprising all the relevant authorities (e.g. health professionals, health informaticians, members of the public, government representatives and law experts) who will contribute to the EHR implementation.
- The government should allow the developed committee to be the principle project owner for EHR implementation as a way of ensuring that political changes do not affect government’s commitment to the project.
- The committee should engage members of the health care community in workshops in identifying the points of resistance where commitment to the utilisation of implemented EHRs may be lost.

### Critical success factor 3: Investigate and invest in alternative infrastructure facilities

This CSF would solve infrastructural challenges of poor Internet connectivity as Internet connectivity is an essential element of ensuring that an EHR system functions properly. Health care centres in the marginal areas of South Africa where Internet connectivity is not available should invest in alternative forms of connectivity solutions, which might provide a solution to the connectivity challenges. Monawe et al.^5^ suggested investigating the possibility of alternative connectivity sources as a way of solving bandwidth problems. While old technology such as 2G and 3G are becoming obsolete, technologies such as these may be sufficient for the transmission of medical data from one health care provider to the other. Repurposing the old equipment that was used for providing these services may be a cost-effective way of improving Internet availability in rural areas.

However, in order to achieve this, there is a need to investigate the currently available technologies and infrastructure, determining their capabilities and expectations that can be made of the technologies. A similar notion was discussed by Akanbi et al.,^4^ who noted that an evaluation of currently available ICT resources is an essential step towards solving infrastructural issues with the implementation of EHR in Ghana.

#### Recommendations regarding critical success factor 3

The issue of a lack of suitable infrastructure to support the EHR implementation continues to be a major challenge, and with the cost of this infrastructure being high, it is necessary to look for alternative, cost-effective solutions. Recommendations for alternative technologies may include the following:

- The government should collaborate with Internet service providers and work on repurposing some of the now obsolete technologies and hardware such as General Packet Radio Service (GPRS), 2G and 3G technologies that are being phased out from more prominent cities in favour of 4G technologies.
- Investigating the feasibility of batch update systems, rather than real-time updating systems, will help in some areas that have poor connectivity.
- The government can invest in solar powered and industrial diesel-powered electricity generators to provide electricity in areas where electricity supply is hard to reach.

### Critical success factor 4: Allocating separate budgets for e-health projects

This factor would address issues related to the cost of implementation as well as inadequate budgetary commitments from governments. The high costs associated with the implementation of EHR systems in public hospitals and clinics may result in them not being able to afford to maintain the EHR from the budget that is allocated for them to run day-to-day operations. The allocation of a separate budget by the government specifically for the implementation of EHR systems as well as other e-health systems will make it more affordable for public hospitals and clinics. Akanbi et al.^4^ noted that proper budget allocation for the implementation of e-health projects is an essential step towards ensuring project success. Without a dedicated budget for the implementation of EHR systems, the implementation of these systems would have a high probability of failing, partly because of the high costs of implementing and maintaining EHR systems.

#### Recommendations regarding critical success factor 4

The issue of costs associated with the implementation of EHRs has been highlighted in the reviewed literature as a barrier to the successful implementation of EHRs in public health. Some of the recommendations that this study makes for government to ensure that EHR implementation, as well as other e-health projects, receive adequate funding include the following:

- The government should allocate a budget specifically for e-health projects such as EHR implementation.
- In line with recommendations given for CSF 2, the administration of the budget fund for e-health should be managed by the committee of individuals selected to plan and coordinate the implementation of e-health projects such as EHR implementation.

### Critical success factor 5: Developing context-relevant e-health implementation strategies and frameworks

This CSF will address the issue of policies, strategies and frameworks and a lack of implementation strategies that are suitable for implementing e-health services in Africa. Literature suggested the development of national frameworks for the implementation of e-health technologies such as EHR systems as one way of ensuring that all health care providers use the same framework standards and there are no interoperability problems between systems.^23,27,29,30^ This includes developing EHR implementation policies, strategies as well as frameworks that take into consideration all the issues and challenges which are unique to South Africa. Following existing frameworks and strategies that were developed based on developed countries that have advanced architectures may result in the implementation not yielding the expected results. Creating professional bodies dedicated to the creation of frameworks and strategies that are tailored to the African context was discussed as a possible solution to the lack of clarity and precision found in some e-health strategies in African countries.^25^

#### Recommendations regarding critical success factor 5

Some of the failures in implementing EHRs in African countries can be attributed to these countries following implementation strategies that are not suited to lowly resourced African countries. Development of implementation strategies that are feasible for African countries is a necessity for ensuring that EHR implementation is successful. This study recommends the following regarding the issue of developing e-health implementation strategies designed for the African context:

- Government should embark on an in-depth investigation of the currently available resources and develop an implementation strategy that is feasible with the available resources.
- Government should commission professional bodies to investigate the most feasible EHR implementation strategies.
- The same professional bodies should be tasked with the development of frameworks for designing of EHR systems that can be implemented using their recommended strategies.

### Critical success factor 6: Develop and implement legislation specific to electronic health record implementation and continued use

This factor will address the issue of legislation that is specific to the implementation and use of EHRs. Developing legislation that governs how the EHR systems can be used can help improve society’s acceptance of the systems. Assuring patients of the safety and privacy of their personal health information will reduce the resistance of the public. Literature suggested that the development of EHR systems can ensure that software vendors conform to a certain standard and their products have interoperability from one vendor to the other.^27^ Legislation can also help with the establishment of service level agreements which will ensure that service providers offer high-quality services and products.

#### Recommendations regarding critical success factor 6

The lack of legislation governing the use of EHRs allows for potential misuse of people’s medical data. It is necessary to implement legislation that will govern the issues of ownership, privacy and security of the medical information collected in EHRs. The following recommendation was made in this study to achieve this.

- The government can facilitate the engagement of experts in the fields of law and health informatics to establish the type of information that will be collected and devise ways of ensuring that legislation is developed that secures such information.

## Conclusion

This study sought to identify CSFs for the implementation of EHRs in South Africa’s public health care sector. The need to implement EHRs was identified in the NHI white paper that was presented by the National Department of Health in South Africa. The National Department of Health highlighted that, for the NHI to be implemented successfully, there was a need to register and track patients as they moved from one health care provider to another.

This study then sought to identify possible challenges that might hinder the implementation of EHRs in South Africa’s public health sector. This study achieved this by reviewing literature regarding EHR implementation in other African countries. Fifteen papers were identified highlighting challenges faced in African countries while implementing EHRs and other e-health services. Three major barriers were identified before CSFs and recommendations to mitigate these problems were discussed.

One of the limitations of this study was the unavailability of literature reviewing implementation issues specific to EHRs in Africa. Most of the articles analysed, while offering relevant information, identified factors that could apply to e-health technology implementation in general. This, however, was an indication that either most African countries had not yet addressed these basic facilitating requirements for implementing e-health technologies or no adequate research had been carried out to evaluate these implementation barriers. A further limitation is the lack of external input on the themes and CSFs that were identified in this article. Future research should evaluate the CSFs and recommendations on the ground through participation of health workers and other experts in the field of health informatics.
